# Medical comorbidities in bipolar disorder (BIPCOM): clinical validation of risk factors and biomarkers to improve prevention and treatment. Study protocol

**DOI:** 10.1186/s40345-024-00337-8

**Published:** 2024-05-04

**Authors:** Giovanni de Girolamo, Ole A. Andreassen, Michael Bauer, Paolo Brambilla, Stefano Calza, Nicholas Citerà, Rosa Corcoy, Andrea Fagiolini, Miguel Garcia-Argibay, Ophélia Godin, Florian Klingler, Nene F. Kobayashi, Henrik Larsson, Marion Leboyer, Silke Matura, Alessandra Martinelli, Víctor De la Peña-Arteaga, Roberto Poli, Andreas Reif, Philipp Ritter, Linn N. Rødevand, Marta Magno, Elisa Caselani, for the BIPCOM consortium, Maximilian Bayas, Frank Bellivier, Narcís Cardoner Álvarez, Pietro Carmellini, Federico Cevoli, Julia Clemens, Philippe Courtet, Lorena Consoli, Giuseppe Delvecchio, Maja Dobrosavljevic, Bruno Etain, Hendrik Friedrichsen, Adrienne Kelemen, Despoina Koukouna, Eugenia Mato, Dídac Mauricio, Romina Miranda-Olivos, Lisa Möbius, Chiara Moltrasio, Caroline Mohn-Haugen, Isabelle Nuss, Emilie Olie, Agnes Pelletier, Zillur Rahman, Davide Rampi, Jonathan Repple, Eugenia Resmini, Julia Schneider, Elena Toffol

**Affiliations:** 1 Unit of Epidemiological and Evaluation Psychiatry, IRCCS Istituto Centro San Giovanni di Dio Fatebenefratelli, Brescia, Italy; 2 Norwegian Center for Mental Disorders Research (NORMENT), Division of Mental Health and Addiction, Institute of Clinical Medicine, Oslo University Hospital, University of Oslo, Oslo, Norway; 3 Department of Psychiatry and Psychotherapy, Universitätsklinikum Carl Gustav Carus an der Technischen Universität Dresden, Dresden, Germany; 4 Department of Neurosciences and Mental Health, Fondazione IRCCS Ca’ Granda Ospedale Maggiore Policlinico, Milan, Italy; 5 Department of Pathophysiology and Transplantation, University of Milan, Milan, Italy; 6 Department of Molecular and Translational Medicine, University of Brescia, Brescia, Italy; 7 Institut de Recerca Sant Pau (IR SANT PAU), Barcelona, Spain; 8 Department of Molecular and Developmental Medicine, Division of Psychiatry, University of Siena School of Medicine, Siena, Italy; 9 School of Medical Sciences, Faculty of Medicine and Health, Örebro University, Örebro, Sweden; 10 Fondation FondaMental, Créteil, France; 11 CIBER de Bioingeniería, Biomateriales y Nanomedicina (CIBER-BBN), 28029 Madrid, Spain; 12 Deutsche Gesellschaft Für Bipolare Störungen (DGBS) E.V, Hamburg, Germany; 13 Department of Psychiatry, Psychosomatic Medicine and Psychotherapy, Goethe University Frankfurt, University Hospital, Frankfurt am Main, Germany; 14 Univ Paris Est Créteil, INSERM U955, IMRB, Translational NeuroPsychiatry Laboratory, Créteil, France; 15 Department of Mental Health, Psychiatric Unit of Cremona General Hospital, Azienda Ospedaliera di Cremona, Cremona, Italy; 16 Fédération Hospitalo-Universitaire de Médecine de Précision en Psychiatrie (FHU ADAPT), Créteil, France

**Keywords:** Bipolar disorder, Metabolic syndrome, Medical comorbidities, Quality of life, Precision medicine

## Abstract

**Background:**

BIPCOM aims to (1) identify medical comorbidities in people with bipolar disorder (BD); (2) examine risk factors and clinical profiles of Medical Comorbidities (MC) in this clinical group, with a special focus on Metabolic Syndrome (MetS); (3) develop a Clinical Support Tool (CST) for the personalized management of BD and medical comorbidities.

**Methods:**

The BIPCOM project aims to investigate MC, specifically MetS, in individuals with BD using various approaches. Initially, prevalence rates, characteristics, genetic and non-genetic risk factors, and the natural progression of MetS among individuals with BD will be assessed by analysing Nordic registers, biobanks, and existing patient datasets from 11 European recruiting centres across 5 countries. Subsequently, a clinical study involving 400 participants from these sites will be conducted to examine the clinical profiles and incidence of specific MetS risk factors over 1 year. Baseline assessments, 1-year follow-ups, biomarker analyses, and physical activity measurements with wearable biosensors, and focus groups will be performed. Using this comprehensive data, a CST will be developed to enhance the prevention, early detection, and personalized treatment of MC in BD, by incorporating clinical, biological, sex and genetic information. This protocol will highlight the study's methodology.

**Discussion:**

BIPCOM's data collection will pave the way for tailored treatment and prevention approaches for individuals with BD. This approach has the potential to generate significant healthcare savings by preventing complications, hospitalizations, and emergency visits related to comorbidities and cardiovascular risks in BD. BIPCOM's data collection will enhance BD patient care through personalized strategies, resulting in improved quality of life and reduced costly interventions. The findings of the study will contribute to a better understanding of the relationship between medical comorbidities and BD, enabling accurate prediction and effective management of MetS and cardiovascular diseases.

*Trial registration*: ISRCTN68010602 at https://www.isrctn.com/ISRCTN68010602. Registration date: 18/04/2023.

**Supplementary Information:**

The online version contains supplementary material available at 10.1186/s40345-024-00337-8.

## Background

Bipolar Disorder (BD) is a severe, complex genetic disorder with a wide range of clinical presentations and a multifactorial etiology (Grande et al. 2016). It ranks as the 6th highest disease burden worldwide and contributes significantly to the global economic burden. BD is a critical public health concern due to its high prevalence (more than 1% of the world's population), chronic nature, high disability rate, and association with both psychiatric and Medical Comorbidities (MC) (Grande et al. 2016). Patients with BD have an elevated risk of cardiovascular disease (CVD) mortality, resulting in a reduced life expectancy of 10–20 years (Miller and Bauer 2014; Laursen 2011; Roshanaei-Moghaddam and Katon 2009), primarily due to a high level of MC in this population (Roshanaei-Moghaddam and Katon 2009), such as type 2 diabetes, CVD, musculoskeletal disorders, renal disorders, neurological disorders, and metabolic syndrome (MetS) (McIntyre et al. 2007). The heightened cardiovascular mortality in individuals with BD is possibly due to multiple factors. Medications like lithium and valproic acid may lead to weight gain and disrupt glucose metabolism, while second-generation antipsychotics are linked to hyperlipidaemia, insulin resistance, and weight gain (Weiner et al. 2011), nonetheless, despite these associations, long-term treatment with antipsychotic drugs has been correlated with lower mortality rates when compared to individuals who do not use antipsychotics (Tiihonen et al. 2009). Lifestyle factors such as poor diet, inadequate exercise, and higher rates of smoking further compound these risks. As a result, common cardiovascular risk factors like obesity, hypertension, diabetes, and hyperlipidemia are more prevalent among those with BD, amplifying their susceptibility to cardiovascular mortality (Weiner et al. 2011; Swartz and Fagiolini 2012). In addition, MC in BD are associated with unfavourable medical outcomes of this mental disorder such as treatment resistance, mood relapse or cognitive disorders (Bauer et al. 2018). Factors contributing to these higher MC rates in BD include pathogenic links between a variety of risk factors such as pathogenic and biological factors (e.g., metabolic, or immune system disorders, genetic susceptibility), medication exposure, unhealthy lifestyles, underutilization of health services, and co-occurring mental disorders (Keck and McElroy 2003; Carney and Jones 2006; Chengappa et al. 2000; Liese et al. 1998). Furthermore, the interplay of genetic susceptibility and underlying physiological processes, including heightened activation of immunometabolic or endocrine systems in both central and peripheral contexts, contributes to the development of both MetS and psychiatric disorders (Penninx and Lange 2018). MetS is a complex and multifaceted medical condition characterized by a cluster of interconnected risk factors that significantly increase the risk of heart disease, stroke, and type 2 diabetes. These risk factors often occur together and share common underlying mechanisms (Eckel et al. 2005). While there is no single universally accepted definition of MetS, several major health organizations and expert groups have agreed on three out of the five criteria listed in Table 1 required to fulfill a diagnosis of MetS (Alberti et al. 2009). The MetS is significantly more prevalent (by 1.98 times) in patients with BD than in the general population (Vancampfort et al. 2013; Godin et al. 2024; Godin et al. 2014). This high prevalence rate is plausibly explained by a range of multiple factors, including medication side effects, unhealthy lifestyles, and pathogenic and biological factors (Firth et al. 2020). For example, certain antipsychotic medications are associated with weight gain and insulin resistance, which in turn increase the risk of developing MetS. In addition, people with BD experience periodic mood fluctuations that affect their ability to maintain a healthy lifestyle (Penninx and Lange 2018). Physical activity (PA) and sleep patterns play important roles in MetS development. MetS is positively associated with physical inactivity and sedentary behaviour, while healthy sleep patterns benefit cardiometabolic health (Amirfaiz and Shahril 2019; Huang and Redline 2019). It is proven that patients with BD are typically less active, more sedentary, and experience poorer sleep quality (Janney et al. 2014; Brochard et al. 2018). Despite the high MC prevalence in BD, patients often receive inadequate diagnoses and treatment, and comprehensive studies on this topic remain limited, hindering personalized plans and preventive treatments. Neglecting comorbidities in BD management creates relevant obstacles to early and effective interventions for patients. For all these reasons, collaborative efforts between mental health and medical services are essential to enhance diagnosis and treatment, improve quality of life, and reduce comorbidity-related mortality in patients with BD.

**Table 1:** Diagnostic criteria for MetS as agreed in the Consensus Statement published in Alberti et al., 2009

Diagnostic criteria	Threshold values
Waist circumference	≥ 102 cm (M); ≥ 88 cm (F)
Fasting plasma glucose	≥ 100 mg/dL or specific treatment
Blood pressure	≥ 130/85 mmHg or specific treatment
Triglycerides	≥ 150 mg/dL or specific treatment
HDL cholesterol	< 40 mg/dL (M); < 50 mg/dL (F) or specific treatment

## Methods

### Aims

The BIPCOM project aims to enhance personalized treatment for comorbid medical conditions, including MetS, in individuals with BD. The project's objectives include: (1) identifying MetS prevalence and risk factors in patients with BD using Nordic registries, biobanks, and medical records; (2) conducting an exploratory clinical study (ECS) with 400 subjects recruited from 11 centres to study the clinical profile and 1-year incidence of MetS; and (3) developing a Clinical Support Tool (CST) that utilizes clinical, biological, and genetic data to enhance personalized treatment for comorbidities, including MetS, with consideration for sex differences. MetS in patients with BD serves as a 'pilot case' due to its implications for prevention and precision medicine (PM). The project aims to pioneer PM approaches in BD, leading to innovative, individualized care models for implementation in clinical practice (Kupfer 2005): these efforts seek to improve healthcare delivery, patient stratification, disease prediction, and prevention, ultimately enhancing patient care and outcomes.

### The general architecture of the project

The BIPCOM project will combine various methods and approaches. These include a register study, a survey of medical records, and the ECS incorporating the use of actigraphy and Experience Sampling Method (ESM). ESM, an ecologically valid method, provides a comprehensive view of daily life, assessing various constructs like quality of life and psychopathology and psychological mechanisms such as stress-sensitivity and coping. These constructs are challenging to obtain through traditional cross-sectional questionnaires. ESM finds applications in treatment monitoring, ecological momentary interventions, clinical trials, and single-case clinical assessments. Data collection can be simplified using a smartphone app (Verhagen et al. 2016). The project will also include a qualitative study with focus groups to assess the multiple dimensions of MC, seen from the ‘eyes’ of patients, clinicians, and family members.

This comprehensive approach will culminate in the development of a CST that will prove valuable to a diverse range of professionals. The BIPCOM project will last 3 years, but the clinical study will take place over a period of approximately 1 year. In the following sections, we will describe all the components of the project.

### Study design

#### Study 1: the registry study

The consortium, drawing on extensive genetic epidemiology expertise, will leverage Swedish and Norwegian national registers and biobanks with (single-nucleotide polymorphism) SNP-array genotyped samples. It will begin by identifying adult Swedish residents from the Swedish National Patient Register and the Swedish Prescribed Drug Register, with registered treatment contact for BD. A similar process will be implemented with Norwegian registers. Subsequently, the consortium will investigate diagnosed MC, comparing individuals with and without BD. The primary focus of the Swedish and Norwegian teams is to explore the associations between BD onset and MC risk, with a specific emphasis on MetS. Additionally, it will include type 2 diabetes and CVD as relevant outcome variables due to their strong association with MetS. Research outcomes will be determined using a combination of diagnostic data from patient registers and pharmacological treatment details from the prescribed drug register, as shown in Table 2. Furthermore, risk prediction models for cardiometabolic conditions in individuals with BD will be developed, with CVD and type 2 diabetes as primary outcomes, and MetS, and all-cause and cause-specific mortality, as secondary outcomes. Given the absence of complete MetS diagnostic criteria in Swedish population registries, the consortium will use several related medical conditions as proxies (e.g., obesity, hypertension, hyper/dyslipidaemia). Access to genotyping data from approximately 1.3 million participants, derived from Illumina arrays or similar platforms in existing genotyped cohorts, will further enrich the study.

**Table 2:** Socio-demographic and clinical characteristics to be used for the training set with the Swedish registry

1	Sex
2	Ethnicity
3	Age
4	Marital status
5	Children (yes/no)
6	Education years, Mean (SD)
7	Area of residence (urban/rural)
8	Current occupational status
9	Poverty (poor housing, malnutrition, etc.)
10	Age of first contact with mental health services (Years), Mean (SD)
11	Bipolar disorder I or II
12	Comorbidity with other mental disorders
13	Number of lifetime psychiatric hospitalizations
14	The overall length of psychiatric hospitalizations (months)
15	Lifetime substance or alcohol use disorder (yes/no)
16	Ever treated with lithium or mood stabilizers
17	Ever treated with antipsychotics
18	Number of diagnoses of MC lifetime
19	Number of lifetime hospitalizations in any medical wards
20	Body Mass Index (BMI)
21	Information about all drug prescriptions in the lifetime
22	Smoking (yes/no)
23	Sleep problems
24	Familiarity with diabetes and/or CVD

While leveraging national registers offers significant advantages, several limitations will have to be considered when interpreting findings from this study. First, the reliance on proxy conditions to represent MetS may introduce potential misclassification bias. The absence of complete diagnostic data in the registries limits the study's ability to accurately capture the true prevalence of MetS among individuals with BD. Additionally, individual proxies might not fully capture the complex interplay of factors contributing to MetS, potentially underestimating the true association between MetS and these outcomes in BD. Second, the study is inherently limited to information available in the registers, which may lack granularity around certain lifestyle factors, clinical metrics, and disease progression markers over time. Lastly, the utilization of specific-cause mortality outcomes should also be interpreted with caution due to potential discrepancies between recorded and actual causes of death (Brooke et al. 2017).

#### Study 2: the study of medical records

BIPCOM offers a unique opportunity to gather and analyse extensive datasets from patients with BD utilizing local medical services across five countries. All centres participating to this project use electronic patient records: an ad hoc Medical Record Abstraction Tool (MRAT) will be employed to extract clinical data and information related to 32 predefined variables (Additional file 1: Table S1), including past and current comorbidities and treatments, and a set of biomarkers related to MetS. Patients with BD will be randomly selected from those with inpatient or outpatient visits, aiming for a total sample size of at least 1,500 patients (300 per site). Rigorous measures will be taken to minimize selection bias and ensure data quality, following Jansen's guidelines (Jansen et al. 2005), which involve cross-verification with source documents, close collaboration with local physicians, written guidelines for data collectors, interobserver studies for training, regular data collector meetings, and random checks of completed MRATs against original medical records. Consent is not required as the data used are fully anonymized clinical routine data. MRAT will capture information on various medical comorbidities, including MetS, diabetes mellitus, dyslipidaemias, obesity, endocrine imbalances, CVD, other significant comorbidities, and concurrent prescribed drug use.

#### Study 3: the Exploratory Clinical Study (ECS)

##### Participants

Study 3 consists of the ECS conducted in five countries (France, Italy, Germany, Norway, and Spain), with a total of 11 recruiting sites (Additional file 1: Table S2). A total of 400 participants, aged 18–65 years, diagnosed with either BD I or BD II or BD not otherwise specified (NOS) according to the DSM-5, will be enrolled: these participants will be selected from patients with BD who have had at least one contact with the mental health service in the past year. Table 3 shows the inclusion and exclusion criteria. To ensure a representative sample of patients with BD, a specific stratification will be employed, according to the following schema: age will be split into two strata (18–45 and 46–65 years), sex will be split into two strata (male and female) and age of onset (before 21 years and after 21 years) will be used as a proxy of disorder severity.

**Table 3:** Inclusion and exclusion criteria for the ECS

Inclusion criteria	Exclusion criteria
Age 18–65 years	Plan to relocate in the subsequent year
Primary diagnosis of BD I or BD II or BD NOS	Severe psychiatric comorbidities (schizophrenia spectrum disorders)
At least one contact with the mental health service in the last year	Severe cognitive impairment
Signed informed consent	Severe substance/alcohol misuse as quantified by specific scores on the Alcohol Use Disorders Identification Test (AUDIT) (Babor et al. 2001) and the Drug Abuse Screening Test (DAST) (Skinner 1982)

The study foresees 2 evaluation times: the first at the entry into the study (T0) and the second after 1 year (T1).

##### Recruitment strategy

A random selection of eligible patients will be conducted within each stratum. Considering an anticipated refusal rate of approximately 30%, we will screen 130 eligible patients per country; the Spanish site will screen about 50 patients and will recruit not more than 30 patients. Trained researchers will conduct assessments using the Structured Clinical Interview for DSM-5 (SCID-5)—Clinical Version, specifically focusing on the BD section, to confirm the BD diagnoses.

##### Participant retention strategies in study 3

Due to the characteristics of the study population group (people with BD, possibly of working age), we have allowed for a 20% dropout rate. To maximise response and participation in the ECS, we propose a reimbursement of transport costs for everyone who decides to participate to the study, based on the availability of funds and the permissions of each ethics committee. Participants may be contacted using several methods of communication (post/phone/email); contact details of all participants, including general practitioner (GP) and clinician details, will be recorded in a ‘keeping in touch’ form and each contact, or contact attempt, made with participants will be recorded in a bespoke contact log. All participants will remain in the study and follow-up data will be sought unless consent for participation in data collection is explicitly withdrawn.

##### Clinical measures

A "Patient Schedule" (PS) will be created to capture socio-demographic, clinical, and treatment-related data of each participant at baseline (T0) and during the 1-year follow-up (T1). Table 4 provides detailed information about all assessment instruments to be administered to participants at T0.

**Table 4:** List of standardized assessment tools in the ECS

Name	Description	Item	Scoring range	Languages of the project in which it was validated
Structured Clinical Interview for DSM-5 (SCID-5)—Clinical Version (First 2014)	Structured clinical interview	Only the BD section		IT, FR, DE, NO, ESP
Alcohol Use Disorders Identification Test (AUDIT) (Babor et al. 2001)	Screening tool to assess alcohol consumption patterns and alcohol use disorders	10	0–40	IT, FR, DE, NO, ESP
Drug Abuse Screening Test (DAST) (Skinner 1982)	Self-report questionnaire to screen for substance use disorders	10	0–6 +	IT, FR, ESP
World Health Organization Disability Assessment Schedule 2.0, self-report version (WHODAS 2.0) (Ustün et al. 2010)	Tool to assess the impact of health conditions on functioning in six domains of life (cognition, mobility, self-care, getting by, activities of daily living, and participation)	12	0–100	IT, FR, DE, NO, ESP
Functioning Assessment Short Test (FAST) (Rosa et al. 2007)	Self-report questionnaire to assess functional impairment and disability in individuals with mental disorders	24	0–40 +	IT, FR, DE, NO, ESP
Elixhauser Comorbidity Index (ECI) (Fabbian et al. 2017)	System for classifying medical comorbidities according to ICD categories	NA	NA	IT, FR, DE, NO, ESP
EQ5D Health questionnaire (Rabin and Charro 2001)	Self-report tool to assess the quality of life focusing on 5 dimensions: mobility, self-care, usual activities, pain/discomfort, and anxiety/depression	5	0–100	IT, FR, DE, NO, ESP
Short Form Health Survey (SF-36) (Brazier et al. 1992)	Self-report tool to assess the quality of life covering eight health-related domains	36	0–100	IT, FR, DE, NO, ESP
Pittsburgh Sleep Quality Index (PSQI) (Buysse et al. 1989)	Self-report tool to assess sleep quality and structure in adults by measuring seven domains	19	0–21	IT, FR, DE, NO, ESP
Childhood Trauma Questionnaire (CTQ) (Bernstein et al. 1994)	Self-report tool used to assess childhood trauma experiences	28	5–125	IT, FR, DE, NO, ESP
Fagestrom scale (Heatherton et al. 1991)	Self-report tool used to assess nicotine dependence in individuals who smoke cigarettes	6	0–10	IT, FR, DE, NO, ESP
Mini-Mental State Examination (MMSE) (Folstein et al. 1975)	Screening tool used to assess cognitive impairment	11	0–30	IT, FR, DE, NO, ESP
Handgrip squeeze test (Sayer et al. 2006)	It measures the maximum isometric strength of the hand and forearm muscles			
Knee extension test (Bohannon 1986)	It measures the hamstring muscle length and the range of active knee extension in the position of hip flexion			

Assessments will be conducted through participants' illness history and BD treatments. A structured physical examination, based on the Physical Examination Essential Checklist (Additional file 1), will be administered, with any signs of ill health promptly reported to clinicians.

During the physical examination, patients will complete the Knee extension test (Bohannon 1986) and the handgrip squeeze test (Sayer et al. 2006) measured through a dynamometer. Additionally, clinical electrocardiograms (ECGs) will be performed to identify QTc prolongation or the presence of arrhythmias. The primary aim of the ECS is to identify common associations among the five MetS diagnostic criteria in patients with BD at both T0 and T1 using clustering techniques and multiple correspondence analyses. Each stratum within each site will include approximately 14 subjects, totaling approximately 57 patients from the 400-sample pool, ensuring data representativeness and statistical power. The 1-year follow-up phase will begin immediately after the baseline assessment. At the end of this period, participants will undergo reassessment using a set of tools (Additional file 1: Table S3) to evaluate the clinical progression or improvement in MetS criteria, focusing on changes among patients meeting these criteria.

##### Biological measures

MetS is defined based on clinical and laboratory measurements (Alberti et al. 2009) and is strongly associated with metabolic liver disease, considered by some authors as an additional component of the syndrome (Kotronen et al. 2009; Kotronen and Yki-Järvinen 2008). There is a myriad of biomarkers associated with MetS that are either included in the pathway to its development and can help in its prediction or are potential mediators of its outcomes. They have been categorized into cardiometabolic, dyslipidemias, markers of oxidative stress, and inflammatory markers (Robberecht and Hermans 2016). Cardiometabolic biomarkers include insulin resistance, thyroid function, and cortisol levels, with the latter two being particularly relevant for patients with BD (Chakrabarti 2011; Dziurkowska and Wesolowski 2021). Adipokines, including adiponectin and leptin, are significantly associated with MetS, with its ratio being proposed as a biomarker of adipose tissue dysfunction (Lee and Shin 2020). Dyslipidemias involve elements additional to those required for MetS definition (Robberecht and Hermans 2016). Furthermore, oxidative stress markers like oxidized low-density lipoprotein (OxLDL) are linked to MetS and its consequences (Holvoet et al. 2008). Inflammation plays a crucial role in MetS, with markers such as high-sensitivity C-reactive protein (hsCRP), serum amyloid A, and cystatin C being associated (Robberecht and Hermans 2016; Magnusson et al. 2013; Ridker 2007). A systematic literature review (Srikanthan et al. 2016) has informed the selection of various biomarkers for MetS, including additional analytes to those mentioned so far: pro-inflammatory cytokines (interleukin-6—IL-6, Tumour Necrosis Factor-alpha—TNF-α), markers of pro-oxidant status (OxLDL, uric acid), and leptin, which are increased in MetS. There is limited available information on most of these biomarkers in this population, as well as their relationship with psychometric variables both cross-sectionally and longitudinally. During the ECS, patients will also be asked to undergo two blood and two saliva samples, one at T0 and one at 1-year follow-up (T1): 24 selected measurements and biomarkers, shown in Table 5, will be collected, shipped to a centralized laboratory, and assessed.

**Table 5:** List of biomarkers to be assessed in the ECS and their normal ranges of values

Adiponectin/leptin ratio	According to the lab reference values
Core biomarkers	
Alanine transaminase (ALT)	< 55 U/L
Aspartate transaminase (AST)	< 34 U/L
C Peptide	0.81–3.85 ng/mL
Fasting plasma glucose	70–99 mg/dL
Gamma-Glutamyl Transferase (GGT)	< 73 U/L (males); < 38 U/L (females)
High-density lipoprotein (HDL)-cholesterol	≥ 40 mg/dl (males); ≥ 50 mg/dl (females)
hsCRP	≤ 0,3 mg/L
Oxidized LDL	20–170 ng/mL
Triglycerides	< 150 mg/dL low risk; 150–199 mg/dL moderate risk; 200–499 mg/dL high risk; > 500 mg/dL very high risk
Additional biomarkers	
Albumin	3.4–5.0 g/dL
Cystatine-C	0.56–0.95 mg/L
IL-6	< 4,4 pg/mL
LDL-cholesterol	< 55 mg/dL very low risk individuals; 55–70 mg/dL low risk individuals; 70–100 mg/dL moderate risk individuals; 100–116 mg/dL very high-risk individuals; > 116 mg/dL extreme risk
Salivary cortisol	Morning: 0.32–10.76 ng/mL Evening: 0.06–2.74 ng/mL
Serum amyloid A	< 6,4 mg/L
Testosterone	Males: 1.97–6.69 ng/mL (< 50 years); 1.87–6.84 ng/mL (≥ 50 years). Females: 0.08–0.35 (< 50 years); 1.87–6.84 (≥ 50 years)
Thyroid antibodies (TPOAb)	< 60.0 U.I./mL
Thyroid antibodies (TGAAb)	< 0.9–4.1 U.I/mL
TNF-α	4.6–12.4 pg/mL
Total cholesterol	< 200 mg/dL
Thyroid-stimulating hormone (TSH)	0.55–4.78 micUl/ml (> 18 years)
Uric acid	3.5–7.2 mg/dL (males); 2.6–6.0 mg/dL (pre-menopause females); 3.5–7.2 mg/dL (post-menopause females)

##### Digital measures

During the ECS, we will employ a digital tool to measure PA and sleep patterns. We will compare about 100 patients who meet (MetS +) or do not meet (MetS−) MetS diagnostic criteria at baseline (about 20 per country) with 100 psychologically healthy control subjects (about 20 per country), matched for both age (by 10-year age group) and sex. Healthy controls will be interviewed to obtain sociodemographic data and information on health status, specifically regarding psychological, cardiovascular, or metabolic diseases. Patients with BD and healthy controls will wear ActiGraph GT9X Link or ActiGraph GT3X devices three times during a year, each time lasting 1 week totalling 3 weeks over a year to assess total PA, intensity-specific PA, sedentary time, and circadian rhythms. In both groups, we will explore associations between PA and different variables, (such as sedentary time, sociodemographic characteristics, and clinical markers) using generalized linear mixed models (GLMM). These models will consider wear time, various activity levels, heart rate, inter-daily stability, intra-daily variability, and rhythm amplitude as factors. Additionally, PA and circadian rhythm variables will predict changes in MetS components between baseline and follow-up assessments. In the same week during the measurement via ActiGraph, the same subgroup of patients undergoing PA monitoring will also complete a dietary assessment using the MyFood24 web portal. Furthermore, the ECS includes an ESM study, prompting patients eight times daily via smartphone to report mood, stressors, eating behaviour, and other variables. This signal-contingent reporting will be complemented by event-contingent reports of eating episodes. This approach will ensure comprehensive data collection in natural settings, will avoid lengthy retrospective self-reports, and will capture participants' environmental, social, and psychological states.

### Data analyses

#### Data quality management

Data quality management and storage are critical aspects of any biomedical study. These processes ensure that the data collected during the study are accurate, reliable, and securely stored for analysis and future reference. In order to guarantee appropriate data quality management, we will adopt the following steps:

(i) Data collection protocols: we will develop well-defined data collection protocols to ensure consistency in data gathering. These protocols may include standardized procedures, guidelines, and training for data collectors to minimize errors and biases.
(ii) Data validation: we will use validation procedures to check the integrity and accuracy of the data. This involves cross-verifying data entries, identifying outliers, and resolving any inconsistencies or missing values.
(iii) Data cleaning: data cleaning involves the process of correcting errors, removing duplicates, and addressing missing or erroneous data points. This step is essential for ensuring the reliability of the final dataset.
(iv) Quality control: regular quality control checks will be performed throughout the study to identify and rectify any potential issues with data collection or recording. These checks will help maintain data accuracy and completeness.
(v) Data monitoring: continuous monitoring of the data collection process will allow study researchers to identify any deviations from the established protocols and take corrective actions promptly.
(vi) Data auditing: an independent review or audit of the data can be performed to validate the accuracy and compliance of the data with study protocols and ethical guidelines.

#### Study 1: the registry study

For prediction modelling studies in Sweden, we will employ multivariate Cox proportional hazards regression analysis to develop 1-, 2- and 5-year risk prediction models separately for: CVD, and type 2 diabetes, as primary outcomes, as well as for proxy measures of MetS—hypertension, obesity and hyper/dyslipidaemia, and all-cause and cause-specific mortality as secondary outcomes, in individuals with BD. This will be done by combining traditional risk factors of cardiometabolic disorders (i.e., age, sex, a diagnosis/medication prescription for hypertension, diabetes mellitus (type 1 and type 2), hyperlipidaemia, obesity, and smoking; and family history), with potentially relevant novel predictors associated with both BD and an increased risk of cardiometabolic conditions (i.e., psychiatric comorbidity, use of psychotropic medication, and socio-demographic factors). We will use a limited backward stepwise procedure to determine whether to retain novel candidate predictors based on their p-values. Traditional risk factors will be kept in the model. With this approach we aim to retain predictors considered as traditional cardiometabolic risk factors based on clinical practice and previous studies, and to consider potentially relevant novel risk factors, with the final model being easily applicable by clinician, with satisfactory face validity. A bootstrapping method will be used for internal validation of the models with 200 bootstrap samples. To assess the discrimination of the models, the ROC curve and C-index will be used. To assess the calibration of the models we will use the Integrated Brier Score and calibration plots. We will also compare the performance of the models that includes only traditional risk factors with the models that include additional novel risk factors by calculating the Net Reclassification Index (NRI) and the Integrated Discrimination Improvement (IDI) index. We will also test the performance of the models across the following subgroups: males/females, and aged younger than 50/50 and older. Machine Learning (ML) algorithms for survival analysis will be tested on Swedish and Norwegian register data, considering a large number of available potential risk indicators such as demographics, electronic medical records, and genotypes. The overarching research question is to explore if ML algorithms based on many predictors may improve the predictive accuracy. For the ML algorithms, the data will be split into a 60% training set, 20% validation set, and 20% test set. Four models will be trained: penalized Cox regression, random survival forest, gradient boosting machine, and survival support vector machine. Hyperparameters will be tuned on the validation set. Model discrimination will be evaluated with C-index and cumulative/dynamic AUC. Calibration will be assessed with the Integrated Brier Score. Performance metrics on the test set will determine the final model. Sensitivity analyses will explore model accuracy when considering predictors related to patient history and dispensed medications only. We will also assess model performance separately in Sweden and Norway. Furthermore, we will merge register data from Norway and Sweden with biobank data (N ~ 1.3 million) to evaluate whether adding genotypes improves predictive power.

#### Study 2: the study of medical records

First, the anonymized data will be collected using a list of 32 predefined items (see Additional file 1: Table S1), some of which are similar to the variables used in the registry study. These data will then be aggregated and presented descriptively. In the second step, the data will be compared with analogous data from all centres included in the study to identify regional differences. Finally, the analysis will be made by comparing this data with corresponding samples gathered from the general population as well as the ongoing H2020 projects REALMENT (grant agreement No. 964874) and TIMESPAN (grant agreement No. 96538). The aim is to identify differences between the study population and the general population.

#### Study 3: the exploratory clinical study

Following the sign of informed consent, we will assess at baseline, the distribution of all possible combinations of the 5 MetS criteria (32 dispositions with repetition of two elements) among BD patients. A subject scoring positive at 3 criteria out of 5 will be defined as MetS + . At baseline, MetS + subjects will be compared to MetS—(less than 3 out of 5 criteria) for 15 predefined items, using either t-test for independent samples (continuous items) or Fisher Exact test (for binary items). Due to test multiplicity, individual tests p-value will be adjusted using Benjamini–Hochberg procedure.

The evolution in MetS status (MetS + /MetS−) from baseline to 1 year follow-up will be evaluated using multistate models, in order to identify what items might affect the transition probabilities. The 5 MetS criteria combinations will be explored using multiple correspondence analyses in order to identify possible clusters based on the predefined items. All the analyses will be performed using R (R Foundation, version 4.3.1 or newer). All tests will be two-sided.

### Power and sample size

#### Study 1: the registry study

To determine the required sample size for fitting a Cox proportional hazards model, recent guidelines were followed (Riley et al. 2019). With an estimated cohort of 40,000 incident patients with BD and an expected 12,000 outcome events based on a 31% prevalence rate (Engin 2017) the minimum required sample size was estimated to be 4,199 individuals for a model with 24 candidate predictors. This assumed an average 2-year follow-up, a shrinking factor of 0.90, and an anticipated R2 of 0.05. For the ML models, sample size calculations based on statistical power are not directly applicable, since these techniques focus on optimizing predictive performance (i.e., reducing a loss function) rather than hypothesis testing. Instead, sample size and model complexity will be considered when training (e.g., learning curves plots by sample size and several parameters).

With a large cohort of 40,000 patients, our study is adequately powered to develop and validate robust predictive models for MetS in patients with BD using both standard survival analysis and ML techniques. This will allow the identification of modest predictive effects across a range of candidate variables and the construction of accurate predictive models for MetS incidence in this population (Engin 2017; Peduzzi et al. 1995).

#### Study 2: the study of medical records

Considering a confidence level of 95% and a standard error of 5%, the study would require the inclusion of at least 385 patients to provide a valid representation of the population under investigation. However, our study takes on an additional layer of complexity as we aim to make a cross-country comparison, analysing differences within the study population. To accomplish this and maintain robust statistical power, we need to consider a power level of 0.9 and an alpha level of 0.05. These choices are essential to ensure that our study is adequately powered to detect any meaningful differences between the countries being examined. Given these criteria and assuming an effect strength (η2) of 0.015, we should aim to include a minimum of 346 patient records from each site, thus totalling 1,730 patient records for the four countries comparative analysis (France, Germany, Italy, Norway, and Spain). This larger sample size is necessary to account for the population differences and to draw meaningful conclusions about the healthcare conditions and outcomes in each country.

#### Study 3: the exploratory clinical study

We estimated the sample size based on planned comparisons for 15 variables of interest (Table 6) for a two independent groups design (MetS + vs MetS−) assuming an expected proportion of Mets (at least three criteria out of five) of approximately 31%. With the estimated cohort of 400 subjects (180 Mets + , 220 Mets−), assuming a power of at least 80% and a significance level of 0.002 (to account for test multiplicity), we are going to be able to identify, at baseline, a significant effect size (ES) of 0.4 for independent t-tests (continuous variables) or an ES of 0.395 for a comparison of two independent proportions (assuming normal asymptotic test).

**Table 6:** List of variables to be used for power calculation in the ECS at baseline

Sex	1. Female, male
Age	1. 18–45 years, 46–65 years
Marital status	1. Married, unmarried
Children (yes/no)	1. Yes, 2. No
Education years, mean (SD)	1. 0–10, > 10
Current occupational status	1. Employed, 2. Unemployed
Age of first contact with mental health services (Years), Mean (SD)	Continuous variable
Bipolar disorder I or II or NOS	1. Yes, 2. No
Comorbidity with other mental disorders	1. Yes, 2. No
Lifetime substance or alcohol use disorder	1. Yes, 2. No
Ever treated with lithium or mood stabilizers	1. Yes, 2. No
Ever treated with antipsychotics	1. Yes, 2. No
Number of diagnoses of MC lifetime assessed with Elixhauser	Continuous variable range 0–32
Body mass index	Continuous variable
Family history of diabetes and/or CVD	1. Yes, 2. No

## Discussion

### The final output of the project: the clinical support tool

The project's primary outcome is the CST, a risk calculator designed to enhance prevention, diagnosis, and treatment of comorbidities in Patients with BD. The CST evaluates key factors such as BMI, PA, job type, diet, lifestyle choices, medical family history, stress events, and biomarkers. It will categorize patients into low, medium, high, or diagnosed comorbidity risk levels. The CST includes three components: an electronic risk assessment questionnaire for physicians and patients, a best practice paper-based recommendation prompt with comorbidity management guidance a risk graph, and a customized educational sheet outlining the patient's risk level. The beta version of the CST will undergo refinement with input from BIPCOM stakeholders. We will test it with 5–10 patients per country from the ECS who were not used in prediction model development. Initially, we will perform retrospective validation using ECS baseline data to assess MetS prediction. Subsequently, the beta CST will be evaluated by 2 clinicians per country for user interface, data access, and clinical applicability. Importantly, the CST will not suggest therapeutic decisions, which will remain the responsibility of treating clinicians. This novel tool addresses a crucial need for evaluating MetS risk in patients with BD and provides valuable insights for future clinical translation, addressing practical, ethical, and user-related considerations in PM in Psychiatry implementation.

### Patient and public involvement: the qualitative study and focus groups

In the BIPCOM project our aim is to acquire current insights regarding the identification and treatment of medical co-occurring conditions in individuals with BD. This includes understanding the impact of these conditions, both addressed and unaddressed needs, and how healthcare services can assist in their management. We will conduct a qualitative study using Focus Groups (FGs) to achieve this. FGs will be conducted simultaneously in six European sites (Additional file 1: Table S4), with three groups per location. Each recruiting site will run three groups, each consisting of 6–7 participants. Of the three groups, one will include patients with BD, the second one BD clinicians, and the third one caregiver, resulting in a total of 18 sessions. Before starting the group sessions, research questions applicable to all groups will be defined. In each group there will be a moderator and an observer. Data analysis will follow the Framework Method (Gale et al. 2013), involving coding and the development of an analytical framework, according to recommended best practice standards in qualitative research using the Consolidated criteria for reporting qualitative research (COREQ) (Tong et al. 2007) We will use nVIVO software for analysis. The insights gathered from the FGs will be crucial to finalize the CST that offers suggestions to aid clinical decision-making in managing comorbidities in BD. The study results will have significant implications for enhancing prevention, early detection, and effective treatment of comorbidities in individuals with BD. A specific and more detailed study protocol regarding the qualitative research will be submitted separately.

### Data transfer

All transfers of study data will be informed by and comply with the European Parliament and the Council of Europe’s Directive 95/46/EC (GDPR) on the protection of individuals concerning the handling of personal data and on the free flow of such information between EU countries. To ensure the security and integrity of data during such transfer, an appropriate documented standard procedure has been established, spelled out in a Data Management Plan, and will be followed without exception. Any study data that are to be transferred between research sites will be anonymized prior to transfer.

### Data storage

All essential trial records will be securely stored by Istituto Centro San Giovanni di Dio, Fatebenefratelli (IRCCS FBF) (Brescia, Italy), and local sites, adhering to regulatory requirements and ensuring privacy compliance. Access will be limited to authorized personnel with role-based access permissions. Regular data backups and a disaster recovery plan will safeguard data integrity. Long-term archiving will ensure data accessibility for future research and replication.

### Data access and quality assurance

At all recruiting sites, we adhere to local research ethics committee requirements and national/EU laws for collecting, recording, sharing, and securely storing person-identifiable data. Personal information about participants is kept confidential throughout the study and afterward. Data access is limited to authorized personnel and provided to relevant bodies for audit purposes only. After the conclusion of the BIPCOM project, analysis data sets will be made available in accordance with the IRCCS FBF Data Management Policy, ensuring compliance with legal, ethical, and funder requirements for data registration, storage, accessibility, and disposal.

### Strength and limitations of the proposal

This project is novel and innovative in several key ways. BIPCOM adopts a comprehensive approach to studying MetS and other MC in BD, filling a gap in research that typically focuses on specific comorbidities. The BIPCOM project employs a multifaceted approach, combining register studies, ecological momentary assessments, medical record analysis, qualitative studies with FGs, digital measures, as well as extensive genetic epidemiology expertise, large-scale genetic data, and a broad array of other clinical and biological assessments. Case-register studies will uncover the prevalence, course characteristics, and mortality rates among patients with BD, enabling the development of prediction models. ML algorithms and predictive modeling will be employed to analyze the vast dataset, enhancing the understanding of the relationship between BD and MetS. Unlike many existing studies reliant on self-reporting questionnaires, BIPCOM will conduct direct, standardized medical assessments and passive activity monitoring using an actigraphy measurement. This approach will enhance the identification of emerging medical conditions, will clarify their progression, and will aid in identifying potential risk factors. The project includes screening specific biomarkers from biological samples, offering valuable insights into the underlying mechanisms of MC, coupled with data collected during the ECS. This comprehensive approach is designed to provide a holistic understanding of comorbidities in BD patients and to facilitate the development of effective prevention and treatment strategies.

Among the study limitations, we acknowledge the absence of more detailed assessments of target organs with the evaluation of echocardiography and ECG for the heart and ultrasound and elastography for the liver. However, properly conducting these assessments in a multicenter study presents several challenges, especially when faced with financial constraints. The primary concerns revolve around the need for standardization, quality control, and the associated costs; echocardiography is highly operator-dependent, and the accuracy and reliability of its results depend significantly on the skill and experience of the operator. Standardizing the exact protocols for performing and interpreting echocardiography across multiple centers (e.g. positioning of patients, measurement procedures, and criteria for abnormal findings) is challenging. Similarly, ultrasound/elastography assessment of the liver can be equipment and operator-dependent (Fang and Sidhu 2020). The costs of standardizing equipment, protocols, and training across multiple centers can be prohibitive, especially for studies with limited budgets, as BIPCOM, and this has prevented us from the adoption of these assessments.

## Conclusions

BIPCOM aims to guide for enhancing healthcare for patients with BD, influencing clinical practices, decision-making, and regulatory frameworks for various stakeholders, including physicians, healthcare providers, and policymakers. The study will investigate system-based clinical and biological markers to monitor patients with BD conditions and identify potential risks and protective factors for comorbidities. Identifying clear risk factors enables early detection and timely interventions, leading to more effective and personalized care, improving overall patient health, and reducing healthcare costs. BIPCOM will also explore shared pathways for predicting adverse events and advancing targeted diagnostics and treatments. It will contribute to policy-making by informing health service administrators, politicians, professional groups, Non-governmental Organizations (NGOs), and business leaders, providing evidence for future funding and policy changes in line with emerging clinical insights. Additionally, BIPCOM will address social challenges, optimize clinical choices, enhance quality of life, reduce healthcare costs, and training young scientists and clinicians in personalized BD treatment approaches. In conclusion, the BIPCOM project is a commendable effort to address the complex issue of comorbidities in individuals with BD. By combining multidisciplinary research methods, cutting-edge technology, and the involvement of various stakeholders, it aims to pave the way for personalized, effective, and comprehensive healthcare for BD patients. The outcomes of this project will have the potential to not only improve the quality of life of those with BD, but also to inform future healthcare policies and practices in the field of psychiatry and broader healthcare in general.

### Supplementary Information

**Additional file 1: Table S1.** Socio-demographic, clinical characteristics, and biomarkers to be used for the study of medical records. **Table S2.** List of participating and recruiting centres. **Table S3.** List of assessment tools at follow-up. **Table S4.** List of participating and recruiting centres in qualitative research.

## Data Availability

The datasets generated and/or analysed during the current study will be available in the Zenodo repository (see https://zenodo.org/).

## Abbreviations

ALT: Alanine transaminase
AST: Aspartate transaminase
BD: Bipolar disorder
BMI: Body mass index
CST: Clinical support tool
CVD: Cardiovascular disease
ECGs: Electrocardiograms
ECS: Exploratory clinical study
ECs: Etichal committees
EF: Effect size
ESM: Experience sampling method
FG: Focus group
GGT: Gamma-glutamyl transferase
HDL: High-density lipoprotein
HsCRP: High-sensitivity C-reactive protein
IDI: Discrimination improvement index
LDL: Low-density lipoprotein
LMM: Linear mixed models
MC: Medical comorbidities
MetS: Metabolic syndrome
ML: Machine learning
MRAT: Medical record abstraction tool
NRI: Net reclassification index
NGOs: Non-governmental Organizations
PA: Physical activity
PM: Precision medicine
PS: Patient schedule
QOL: Quality of life
SCID-5: Structured clinical interview for DSM-5
TGAAb/TPOAb: Thyroid antibodies
TNF-α: Tumour necrosis factor alpha
TSH: Thyroid-stimulating hormone
