# Supplementary material for: Medical comorbidities in bipolar disorder (BIPCOM): clinical validation of risk factors and biomarkers to improve prevention and treatment. Study protocol
Source: Int J Bipolar Disord. 2024 May 4;12:15. doi: 10.1186/s40345-024-00337-8 (PMC11069492; doi:10.1186/s40345-024-00337-8)
Supplement: Supplementary file 1 — Additional file 1: Table S1. Socio-demographic, clinical characteristics, and biomarkers to be used for the study of medical records. Table S2. List of participating and recruiting centres. Table S3. List of assessment tools at follow-up. Table S4. List of participating and recruiting centres in qualitative research. [file 40345_2024_337_MOESM1_ESM.docx]

**Additional file**

**Physical examination essential checklist**

| **RESPONSE OPTIONS** | **YES** | **NO** | **PARTIAL** |
| --- | --- | --- | --- |
| VITAL SIGNS |  |  |  |
| Measured pulse rate by palpating radial pulse or auscultated at apex of heart at least 30 sec |  |  |  |
| Measured blood pressure in one arm, 2 step for initial measurement (not baseline record):   1. used appropriate size cuff, 2. placed on inch (2cm) above antecubital space, 3. inflated cuff 30mmHg above pulse disappearance (palpate or auscultate), 4. deflated cuff at 2-3mm Hg per sec, until 20-30 below last sound |  |  |  |
| Measured blood pressure in one arm, 1 step for repeat measurement   1. used appropriate size cuff, 2. placed on inch (2cm) above antecubital space, 3. inflated cuff 30mmHg above pulse disappearance (palpate or auscultate), 4. deflated cuff at 2-3mm Hg per sec, until 20-30 below last sound |  |  |  |
| Measured Respiratory Rate- at least 30 sec |  |  |  |
| NECK |  |  |  |
| Palpated carotids   1. at level of thyroid cartilage 2. right and left (not same time) |  |  |  |
| Auscultates Carotids   1. with bell 2. bilaterally 3. with patient breath held |  |  |  |
| CHEST AND PULMONARY EXAM |  |  |  |
| Inspects- chest wall for shape and symmetry |  |  |  |
| Performed percussion of posterior lung fields   1. cephalad to caudal (top to bottom) 2. bilaterally 3. at least three areas (upper lobe to lower lobe) |  |  |  |
| Performed auscultation of anterior lung fields bilaterally   1. upright, seated 2. at two levels, at least (upper lobe and lower) 3. both right and left |  |  |  |
| Performed auscultation of posterior lung fields bilaterally   1. upright, seated 2. at least three areas (upper lobe to lower lobe) 3. both right and left |  |  |  |
| Auscultates lateral lung fields -one area each, right and left |  |  |  |
| CARDIAC EXAM |  |  |  |
| Drapes   1. must be able to listen on skin 2. attends to patient comfort, through draping |  |  |  |
| Inspects   1. precordium 2. neck veins, carotid pulse 3. apex of heart (Left Lower Sternal Border, 5th InterCostalSpace) |  |  |  |
| Palpates heart   1. at apex 2. over right ventricle (Left Lower Sternal Border or epigastric area) 3. at base (Right Upper Sternal Boarder, Left Upper Sternal Border) |  |  |  |
| Auscultates with patient in 3 positions, i.e.   1. upright, seated 2. supine 3. left lateral |  |  |  |
| Auscultates in correct locations (all 4 areas of the heart), i.e.   1. Upper right sternal border (aortic area) 2. Upper left sternal border (pulmonic area) 3. Lower left sternal border (right ventricular area) 4. Apical Impulse (Left ventricular area) |  |  |  |
| Auscultates with both bell and diaphragm (all 4 areas) |  |  |  |
| EXTREMITY EXAM |  |  |  |
| Palpates legs for edema with moderate pressure for 5 seconds |  |  |  |
| PERIPHERAL VASCULAR EXAM |  |  |  |
| Palpates radial pulses, bilaterally |  |  |  |
| Palpates brachial pulses, bilaterally |  |  |  |
| Palpates posterior tibial pulses |  |  |  |
| Palpates dorsalis pedis pulses |  |  |  |

Contributions for this checklist from Kim Tartaglia, Jane Goleman, Cami Curren, Paul Weber, Alan Letson, Julie Bishop, Adam Quick, Mary Beth Fontana, Troy Schaffernocker, Udi Nori, Sheryl Pfeil, Maria Lucarelli.

**Table 1S. Socio-demographic, clinical characteristics, and biomarkers to be used for the study of medical records**

| 1 | Sex | (male/female) |
| --- | --- | --- |
| 2 | Age | Age during the last outpatient/inpatient contact |
| 3 | Marital status | (yes/no) |
| 4 | Children | (yes/no) |
| 5 | Education years | Mean SD & highest educational qualification |
| 6 | Current occupational status | (full-time, part-time, early retirement, retirement, unemployed) |
| 7 | Age of first contact with mental health services | (Years), Mean (SD) |
| 8 | Bipolar disorder I or II | ICD 10: F31.0- F31.9 |
| 9 | Comorbidity with other mental disorders | ICD 10: F-Diagnosis |
| 10 | Number of lifetime psychiatric hospitalizations | N |
| 11 | Overall length of psychiatric hospitalizations | (months) |
| 12 | Lifetime substance or alcohol use disorder | (yes/no); (legal/illegal substances) |
| 13 | Ever treated with lithium or mood stabilizers | (yes/no) |
| 14 | Ever treated with antipsychotics | (yes/no) |
| 15 | Number of diagnoses of MC lifetime | Continous variable based on the Elixhauser Index, range 0-32 |
| 16 | Lifetime MC Diagnosis | According to ICD-10 |
| 17 | Body Mass Index | (kg/m^2^) |
| 18 | Smoking (yes/no) | (yes/no) |
| 19 | Sleep problems | (yes/no) |
| **Biomarkers** | | |
| 20 | Fasting glucose | mg/dl |
| 21 | HbA1c | % |
| 22 | HDL | mg/dl |
| 23 | LDL | mg/dl |
| 24 | Triglycerides | mg/dl |
| 25 | Total cholesterol | mg/dl |
| 26 | TSH | mIU/l |
| 27 | Blood count |  |
| 28 | ALT | U/l |
| 29 | AST | U/l |
| 30 | GGT | U/l |
| 31 | Albumin | g/dl |
| 32 | CRP | mg/l |

**Table 2S****. List of participating and recruiting centres**

| **Country** | **Role in the project** | **Name of Beneficiary** | **Recruiting Centres** |
| --- | --- | --- | --- |
| **Italy** | Coordinating site | IRCCS Istituto Centro San Giovanni di Dio, Fatebenefratelli (IRCCS FBF) (Brescia) | - IRCCS FBF (Brescia) - IRCCS Ca' Granda Ospedale Maggiore Policlinico (Milano) - Azienda Ospedaliero-Universitaria Senese (Siena) - Azienda Socio Sanitaria Territoriale (Cremona) |
| **France** | Partner | Fondation FondaMental (Crèteil) | - Université de Paris - Hôpital LaPeronie (Montpellier) - Hopital Albert Chenevier (Crèteil) |
| **Germany** | Partner | Technische Universität Dresden (Dresden) | Technische Universität Dresden (Dresden) |
| **Germany** | Partner | Johann Wolfgang Goethe-Universität Frankfurt am Main (Frankfurt) | Johann Wolfgang Goethe-Universität Frankfurt am Main (Frankfurt) |
| **Germany** | Partner | Deutsche Gesellschaft für Bipolare Störungen (GSBD) | NA |
| **Norway** | Partner | University of Oslo (Oslo) | University of Oslo (Oslo) |
| **Spain** | Partner | Institut de Recerca de l’Hospital de la Santa Creu i Sant Pau (Barcelona) | Institut de Recerca de l’Hospital de la Santa Creu i Sant Pau (Barcelona) |
| **Sweden** | Partner | Örebro University (Örebro) | NA |

**Table 3S. List of assessment tools at follow-up**

| 1 | World Health Organization Disability Assessment Schedule 2.0, 12-items, self-report version (WHODAS 2.0) |
| --- | --- |
| 2 | Elixhauser Comorbidity Index (ECI) |
| 3 | EQ5D Health questionnaire |
| 4 | Short Form Health Survey (SF-36) |

**Table 4S. List of participating and recruiting centres in qualitative research**

| **Country** | **Role in the project** | **Name of Beneficiary** | **Recruiting Centres** |
| --- | --- | --- | --- |
| **Italy** | Principal Investigator | IRCCS Istituto Centro San Giovanni di Dio, Fatebenefratelli (IRCCS FBF) (Brescia) | - IRCCS FBF (Brescia) - IRCCS Ca' Granda Ospedale Maggiore Policlinico (Milano) - Azienda Ospedaliero-Universitaria Senese (Siena) |
| **France** | Partner | Fondation FondaMental (Crèteil) | - Hopital Albert Chenevier (Crèteil) |
| **Germany** | Partner | University Hospital Carl Gustav (Dresden) | University Hospital Carl Gustav (Dresden) |
| **Germany** | Partner | Johann Wolfgang Goethe-Universität Frankfurt am Main (Frankfurt) | Johann Wolfgang Goethe-Universität Frankfurt am Main (Frankfurt) |
